# Stable Isotopically Labeled Intravenous Microdose Pharmacokinetic Trials as a Tool to Assess Absolute Bioavailability: Feasibility and Paradigm to Apply for Protein Kinase Inhibitors in Oncology

**DOI:** 10.1002/cpdd.840

**Published:** 2020-06-22

**Authors:** Jeroen Roosendaal, Hilde Rosing, Jos H. Beijnen

**Affiliations:** ^1^ Department of Pharmacy & Pharmacology Netherlands Cancer Institute – Antoni van Leeuwenhoek Amsterdam The Netherlands; ^2^ Division of Pharmacoepidemiology and Clinical Pharmacology Science Faculty Utrecht Institute for Pharmaceutical Sciences Utrecht University Utrecht The Netherlands; ^3^ Division of Pharmacology Netherlands Cancer Institute – Antoni van Leeuwenhoek Amsterdam The Netherlands

**Keywords:** absolute bioavailability, isotope, mass spectrometry, microdose, stable isotopically labeled

Absolute bioavailability is defined as a measure of the extent to which the administered drug is absorbed systemically and becomes available in the general circulation compared with intravenously administered drug.^1^ For orally administered drugs, obtaining data on absolute bioavailability is an important component of clinical drug development. Low bioavailability may indicate poor solubility and/or permeability, membrane transport, and/or enzymatic metabolism.^1^, ^2^ Knowledge on absolute bioavailability in an early stage of clinical development is therefore considered essential to allow for the development of optimal drug formulations.

Despite the clear usefulness, absolute bioavailability determination is not mandatory and therefore not a routine part of clinical drug development.^1^ For the group of orally administered tyrosine kinase inhibitors, an important oral drug class in oncology, it was identified that for more than half the drugs registered up to 2014, an absolute bioavailability trial was not performed during clinical drug development.^2^ The main reason for this might be that the assessment of absolute bioavailability requires the formulation and safety testing of an intravenous formulation at therapeutic strength, which serves as a reference to the oral formulation. Technical issues (eg, poor solubility) as well as costs associated with development and safety testing of an intravenous formulation make it often omitted.

The microdose trial design may aid overcoming these problems by making use of an intravenous microdose formulation, defined as less than 1/100th of the therapeutic dose with a maximum of 100 μg. Because microdose studies involve exposure to very small amounts of drug, additional safety testing of the intravenous formulation is not required. Furthermore, drug solubility issues are most often no longer a problem, as only a 100‐μg amount needs to be dissolved into an intravenous formulation.^3^, ^4^ Originally, the major concern with microdosing has been the potential for nonlinear pharmacokinetics between the microdose and the therapeutic dose.^5^ The introduction of stable isotopically labeled microdosing has made it possible to overcome this problem.^6^ By allowing simultaneous administration of a labeled microdose next to a therapeutic unlabeled dose, this new approach has provided opportunity to further improve absolute bioavailability trial designs.

In this review, we describe the way clinical absolute bioavailability trials are conducted using both a conventional trial design and a microdose trial design. The use of a stable isotopically labeled microdose (SILM) in combination with ultrasensitive liquid chromatography–tandem mass spectrometry (LC‐MS/MS) as an analytical technique is described in more detail. For the group of orally administered small‐molecule protein kinase inhibitors (smPKIs), we investigated whether absolute bioavailability was determined during clinical drug development and if a SILM trial design in combination with LC‐MS/MS would have been feasible. We conclude by discussing how the use of SILM studies can affect the execution of absolute bioavailability trials in the future.

## Absolute Bioavailability Trial Design

### Conventional Absolute Bioavailability Trial Design

The absolute bioavailability of novel oral anticancer agents is normally investigated using a 2‐period crossover trial design.^1^ After intravenous and oral administration of the study drug at therapeutic strength during different dose events, exposure as defined by total area under the plasma concentration‐time curve (AUC) is calculated for each dose route. Dividing the equations for the intravenous (iv) and extravascular (ev) administration gives the classical equation for calculating bioavailability, as shown in equation 1:

(1) $$F\;=\left(\frac{\mathrm{AU}C_{\mathrm{ev}}}{\mathrm{AU}C_{\mathrm{iv}}\;}\right)\;\;\times \;\left(\frac{\mathrm{Dos}e_{\mathrm{iv}}}{\mathrm{Dos}e_{\mathrm{ev}}}\right)$$

The conventional absolute bioavailability trial design is limited in 2 ways. First, an intravenous formulation at therapeutic strength is required, which is not always available for drugs that are poorly soluble in aqueous solutions, and, if available, requires additional safety testing. Second, the 2‐period crossover trial design assumes linear pharmacokinetics and equal clearance between the 2 dose events, which is not always the case. A potential way to overcome these problems is provided by the microdosing trial design.^1^

### Microdose Absolute Bioavailability Trial Design

**Figure 1:**
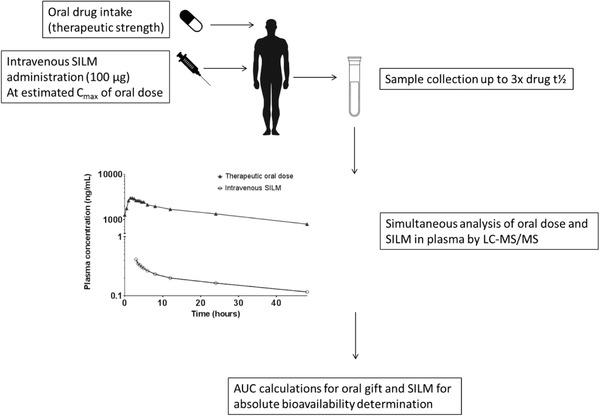
Schematic overview of the stable isotopically labeled microdose trial design to determine the absolute bioavailability of oral anticancer agents.

A microdose is defined as 1/100th of the therapeutic dose with a maximum of 100 μg. In an absolute bioavailability microdose trial, the oral therapeutic dose intended for clinical use is administered, after which the intravenous microdose is administered concomitantly at the estimated maximum plasma concentration of the oral dose (Figure 1). The absolute bioavailability can then be calculated the same way as for the conventional trial design (equation 1). To be able to use this trial design, there is, however, a need to differentiate between the intravenous and extravascular drug exposure to be able to calculate absolute bioavailability. This differentiation can be achieved by using isotope drug labels incorporated into the drug of interest for intravenous administration, using either ^14^C‐radiolabels or stable isotope labels. Depending on the isotope label used, different analytical techniques are required. For ^14^C‐labeled drug, traditional accelerator mass spectrometry (AMS) is most often used, in which the ^14^C:^12^C ratio is calculated, with no regard to the molecular structure of the compound. For quantification of an SILM, LC‐MS/MS is used, in which analysis is based on the mass difference between isotopically labeled and unlabeled drug.

By making use of an intravenous isotopically labeled microdose instead of an intravenous therapeutic dose, solubility issues can be circumvented, as only a maximum of 100 μg of drug needs to be dissolved in an intravenous formulation. In addition, the microdose trial design results in fewer dose events required and in a more accurate calculation of the absolute bioavailability, as all measurements take place in a single subject during a single dosing event, eliminating intrasubject variability and concentration‐dependent clearance.

### Absolute Bioavailability Microdose Trials—Analytical Techniques Involved

Initially, microdose trials were performed exclusively using a combination of AMS and ^14^C‐labeled drug because of the superior sensitivity of this approach (up to the attomole range). Already in the early 1980s, the use of SILMs for clinical pharmacokinetic investigations were discussed by Browne et al.^7^, ^8^ Two major drawbacks of using SILMs in clinical trials at that time were the need for sensitive mass spectrometric analysis equipment and the high costs associated with the purchasing of SILMs.^8^ Recent developments in the field of mass spectrometry led to the development of triple quadrupole LC‐MS/MS assays reporting lower limits of quantification in the pg/mL range, reaching sensitivity levels that before were only possible for AMS.^3^, ^4^, ^9^, ^10^, ^11^ For this reason, SILM in combination with LC‐MS/MS assays now provides an alternative for ^14^C‐radiolabeled drug in combination with AMS to perform clinical microdose trials.

Both LC‐MS/MS and AMS have their own advantages and disadvantages when used as an analytical technique for the support of absolute bioavailability microdose trials. An advantage of using AMS is that ^14^C‐radiolabeled drug material is normally also required for preclinical and clinical absorption, distribution, metabolism, and excretion trials (also known as mass balance trials). In this case, additional synthesis efforts for the intravenous ^14^C‐radiolabeled microdose formulation is not required, which might save time and money. Furthermore, the use of a ^14^C‐radiolabeled microdose does not require additional radiosafety testing, as the extreme sensitivity of AMS requires only minute amounts of ^14^C administered to humans in a clinical study.^1^ Although AMS is also benefited by superior sensitivity compared with LC‐MS/MS, a major drawback of this technique is that it is still very time‐consuming, costly, and analytically challenging, limiting its general practical usability.^12^ For example, chromatographic separation of the ^14^C‐labeled drug molecule from metabolites bearing the ^14^C‐label is required to allow for correct calculation of parent molecule exposure. Some of the technical issues associated with current AMS applications may in the future potentially be overcome by the use of more compact AMS systems with direct liquid chromatography interfacing (LC‐AMS) and automatic sample handling. At the moment, however, these technological improvements of AMS are not yet considered ready for implementation in clinical practice.^13^ Another inherent drawback of using AMS for ^14^C‐radiolabeled microdose analysis is that the calculation of the exposure to unlabeled drug still requires an LC‐MS/MS method next to an AMS method, complicating sample processing and analysis and prolonging study duration.

A major advantage of using stable isotopically labeled drug instead of ^14^C‐radiolabeled drug is that in principle LC‐MS/MS analysis can be used for the simultaneous analysis of both isotopically labeled and unlabeled drug, circumventing the use of 2 different techniques for drug analysis following concomitant intravenous and oral drug administration. A drawback of LC‐MS/MS can be that required sensitivity levels for accurate quantification of plasma drug exposure following intravenous microdose administration cannot be reached. Furthermore, the use of stable isotopically labelled drug for the intravenous microdose may result in a kinetic isotope effect (KIE), caused by increased bond strength of the carbon‐deuterium bond compared with the carbon‐hydrogen bond. The KIE may result in altered pharmacokinetics (eg, altered metabolism) of the stable isotopically labeled drug, with incorrect calculation of the absolute bioavailability as a result.^6^ To prevent this, the potential KIE is ideally investigated in a preclinical setting before the clinical absolute bioavailability trial. In addition, stable isotopically labeled drug with the right amount of isotope labels needs to be synthesized for the sole purpose of the absolute bioavailability study. For each drug, a careful evaluation of primarily LC‐MS/MS sensitivity and selectivity is required before study conduct, which is not required for AMS. However, if considered feasible, the SILM trial design using LC‐MS/MS as an analytical technique results in the most elegant and efficient approach for absolute bioavailability assessments.

### Absolute Bioavailability of Oral Kinase Inhibitors

The group of orally administered smPKIs forms a promising and rapidly expanding class of drugs in oncology.^14^, ^15^ Up to 2019, the US Food and Drug Administration (FDA) has approved 48 smPKIs, of which 41 are orally administered drugs for an oncological indication.^16^ Recently it was identified that for more than half the registered tyrosine kinase inhibitors up to 2014, an absolute bioavailability trial was not performed during drug development.^2^ The majority of drugs for which an absolute bioavailability trial was performed display reduced and variable bioavailability, mainly caused by poor drug solubility and permeability.^2^, ^17^ This variable bioavailability may contribute to significant variation in plasma levels and exposure after oral smPKI intake. Because many oral smPKIs demonstrate large intersubject variability, therapeutic drug monitoring (TDM) in patients treated with these drugs is often required to monitor safety and efficacy during long‐term treatment.^18^ Knowledge on the individual contribution of absolute bioavailability to the variability in plasma drug exposure in an early stage of clinical drug development may help to improve drug formulations and thereby to reduce the need for TDM for some drugs after market approval.

For each oral smPKI granted market approval by the FDA up to 2019, we identified if an absolute bioavailability trial was performed at the time of drug licensing. In case an absolute bioavailability trial was performed, we assessed whether a 2‐period crossover trial design or a microdose trial design was executed. We searched for published articles as well as FDA clinical pharmacology and biopharmaceutics reviews to assess whether these studies have been performed and what type of study and isotope drug label were used to determine absolute bioavailability. If not performed, we identified the reasons for this and investigated the feasibility of using the SILM trial design in combination with LC‐MS/MS to assess the absolute bioavailability.

### Assessment of Stable Isotopically Labeled Microdose Trial Feasibility

There are 2 critical parameters to assess whether a SILM trial approach is technically feasible for the drug of interest. First, a sensitive LC‐MS/MS assay is required with an adequate lower limit of quantification (LLOQ) following administration of a 100‐μg microdose. This was defined as the concentration at 3 times terminal elimination half‐life after administration of 100 μg of intravenous drug. Second, the amount of isotope labels required to differentiate between isotopically labeled and unlabeled drug in plasma after concomitant administration of an oral therapeutic and an intravenous microdose needs to be established. The production of stable isotopically labeled drug with a sufficient amount of isotope drug labels is an important component as well, but the synthesis of stable isotopically labeled drug was outside the scope of this review.

### Determination of Required LC‐MS/MS Sensitivity

To be able to calculate the required LLOQ of the LC‐MS/MS assay, information on certain drug‐specific pharmacokinetic parameters is required. At the start of clinical drug development, phase 1 first‐in‐human trials often provide data on different pharmacokinetic parameters of the drug of interest after administration of a known extravascular dose at the intended therapeutic concentration. Using the apparent volume of distribution (V_d_/F_ss_) at steady state, the theoretical maximum plasma concentration (C_max_) after intravenous administration can be calculated.

Ideally, you would like to be able to measure drug concentrations at least up to 3 times the terminal phase half‐life (t_½_) to be able to accurately extrapolate the area under the plasma concentration‐time curve to infinity (AUC_0‐inf_). The concentration at this time is the C_max_ × 0.5^3^. This is the required LLOQ of the LC‐MS/MS assay to accurately quantify the intravenous SILM up to the last point of collection. Based on available literature, we searched for the V_d_/F_ss_ of the therapeutic extravascular dose at steady‐state plasma pharmacokinetics. Using this parameter, the required concentration of the LLOQ (C_LLOQ_) of the intravenous microdose could be calculated using the following equation:

(2) $$C_{\mathrm{LLOQ}}=\frac{\mathrm{Dose}}{V_{d}/F_{\mathrm{ss}}}\;\times \;0.5^{3}$$

For each smPKI, we calculated the C_LLOQ_ required to perform a SILM trial. Previous research on the clinical relevance of LC‐MS/MS as an analytical tool for the support of microdose studies has demonstrated that LLOQs in the range from 0.08 to 50 pg/mL can be reached for drugs with a variety of molecular characteristics.^11^ Of the 31 drugs investigated in this study, for 27 drugs an LLOQ ≤ 5 pg/mL was obtained using standard sample cleanup methods.^11^ Furthermore, the trial demonstrated that the obtained LLOQs were suitable for the analysis of drug concentrations up to 3 half‐lives for all but 1 analyte following administration of a 100‐μg intravenous microdose. Based on these results, we considered a C_LLOQ_ of 1 pg/mL as an arbitrary threshold for the SILM trial design in combination with LC‐MS/MS to be potentially feasible.

### Determination of Required Amount of Stable Isotope Labels

The amount of isotope labels required to distinguish the intravenously administered SILM from the orally administered unlabeled drug in plasma was calculated using molecular isotopic abundance patterns. For this, we used C_max,ss_ concentrations of the orally administered drug in steady‐state conditions derived from literature. Using the C_max,ss_ for each drug in combination with the isotopic abundance pattern, the amount of naturally abundant isotopic drug originating from the oral dose that could interfere with the intravenously administered isotopically labeled microdose could be determined. In this way we calculated the amount of isotope labels required to selectively quantify the isotopically labeled microdose in the presence of unlabeled drug. The number of isotope labels was deemed sufficient when the interference originating from the oral drug at the C_max,ss_ was calculated to be ≤20% of the C_max_ of the microdose, as defined by general selectivity criteria of European Medicines Agency and FDA guidelines on bioanalytical method validation. The maximum allowed interference was calculated using the following formula:

(3) $$\begin{array}{ccc} & & \mathrm{Max}.\;\mathrm{interference}\;\;\left(\%\right)\\ & & =\frac{\;(C_{\max,\;\mathrm{microdose}\;}\times \;0.2)\;}{C_{\max,\mathrm{ss},\;\mathrm{oral}\;\mathrm{dose}}\;}\;\;\times \;100\% \end{array}$$

In case of ibrutinib, the maximum interference allowed is 0.000905% using a C_max,microdose_ of 0.010 ng/mL and a C_max,ss,oral dose_ of 221 ng/mL. Using the isotope abundance pattern of ibrutinib, the incorporation of 5 isotope labels in the structure would result in 0.0029% interference, whereas the incorporation of 6 isotope labels would result in 0.0001% interference. The minimal required amount of isotope labels in this case is therefore 6.

The use of molecular isotopic abundance patterns for the calculation of the required number of isotope labels can result in overestimation of the actual required number of isotope labels, as the location of the isotope labels also plays an important role in selective LC‐MS/MS analysis. This is because isotopic distribution patterns only account for the complete chemical structure, whereas LC‐MS/MS analysis monitors a transition from the complete chemical structure to a certain fragment. This fragment may or may not contain stable isotope labels, depending on the location of the isotope labels in the drug structure. The method described by Gu et al^19^ and Jiang et al^20^ accounts for the location of the stable isotope labels in the molecular structure. Using the method by Gu et al, the exact isotopic interferences calculated can be less than the calculated interferences based on molecular isotope abundance, as the fragmentation pattern for the latter is not taken into account. To use this technique, information on the location of the labels in the molecular drug structure is required. As we did not have isotopically labeled drug molecules available, the number of isotope labels required using the method by Gu et al was not calculated. However, the number of isotope labels required to perform an unbiased SILM trial will always be the same as or less than the number of isotope labels calculated using molecular isotopic abundance patterns.

## Results

### Absolute Bioavailability Trials Performed for Oral smPKIs

Supplementary Table 1 shows the FDA registered smPKIs up to 2020 for which an absolute bioavailability trial study has been performed and what study design was used. Of the 41 smPKIs, for only half the drugs (21 of 41) was an absolute bioavailability trial executed. The median absolute bioavailability found was 46% (range, 4%‐98%). In case an absolute bioavailability trial was performed, the traditional 2‐period crossover design was still most often used (12 of 20 trials). For 7 drugs an intravenous ^14^C‐radiolabeled microdose design was used. Only for 2 drugs (abemaciclib and ibrutinib) was an SILM approach used.

As seen in Supplementary Table 1, most absolute bioavailability trials were performed in healthy volunteers (16 of 21 trials). For the 5 studies performed in cancer patients, 3 trials were conducted using a ^14^C‐radiolabeled microdose approach (dabrafenib and trametinib) and 2 trials using a 2‐period crossover trial design (gefitinib and pazopanib).

### Stable Isotopically Labeled Microdose Trial Feasibility

The LLOQs of the LC‐MS/MS method required to accurately quantify stable isotopically labeled drug and the minimum number of isotope labels required per drug can be found in Supplementary Table 2. For each smPKI the required LLOQ was calculated to determine plasma levels following a 100‐μg intravenous microdose bolus injection up to 3 times the drug elimination half‐life. Using the arbitrary LLOQ of 1 pg/mL, the SILM design would have been feasible for all 41 investigated drugs. For 26 drugs, an LLOQ ≥10 pg/mL would already be sufficient to perform an SILM trial. For the 2 drugs for which an SILM trial design was used (abemaciclib and ibrutinib), the calculated LLOQ was 18.1 and 1.3 ng/mL, respectively.

The median amount of stable isotope labels required to distinguish the orally administered drug from the concomitant intravenously administered microdose at the estimated maximum plasma concentration of the oral dose was 6 (range, 3‐9). For abemaciclib and ibrutinib, stable isotopically labeled drug containing 8 and 6 isotope labels (abemaciclib‐^13^C_8_ and ibrutinib‐^13^C_6_), respectively, were used.

## Discussion

An important finding of this review is that for more than half the FDA‐registered smPKIs an absolute bioavailability trial has not been performed. A likely explanation for this finding may be that preparation of an intravenous formulation at therapeutic strength might not have been possible because of poor drug solubility. Another explanation may be that, although requested, data on the absolute bioavailability are not strictly required by regulatory agencies for market authorization.

The retrospective analysis of smPKIs registered for oncological indications demonstrates that the SILM trial design provides a useful alternative to the traditional 2‐period crossover trial design, as well as to the ^14^C‐radiolabeled microdose trial design. Technological limitations of the past, mainly caused by insufficient sensitivity of LC‐MS/MS equipment, are becoming less relevant, as it is now possible to quantify drug concentrations in the low pg/mL range.^11^, ^21^, ^22^ In literature, the LLOQs of LC‐MS/MS assays for smPKI analysis in plasma are most often in the low ng/mL range and based on expected plasma concentrations following administration of a dose at therapeutic strength.^23^ For this reason, the published LLOQs might not be reflective of the LLOQs that can be obtained when the limits of sensitivity of LC‐MS/MS equipment are explored.

The SILM absolute bioavailability trials of abemaciclib and ibrutinib demonstrate this finding. In our analysis, we calculated a required LLOQ of 18.1 and 1.3 pg/mL and isotope label number of 5 and 6, respectively, to allow for accurate quantification of the intravenously administered microdose. For these drugs, LC‐MS/MS assays have been developed with an LLOQ of 2 pg/mL for each drug to analyze abemaciclib‐^13^C_8_ and ibrutinib‐^13^C_6_ drug concentrations following microdose administration.^24^ The LLOQs described for the analysis of therapeutic concentrations of these drugs are 0.5 ng/mL for ibrutinib^23^ and 1 ng/mL for abemaciclib,^24^ indicating the potential to improve the sensitivity of LC‐MS/MS assays more than a 100‐fold when there is a direct clinical need. In our laboratory, we recently demonstrated the possibility o use LC‐MS/MS analysis for the support of 2 microdosing trials.^25^ For gemcitabine and imatinib‐d8, fit‐for‐purpose LC‐MS/MS assays were developed with an LLOQ of 2.5 and 10 pg/mL, respectively. These examples also demonstrate the potential of using LC‐MS/MS for the support of clinical microdose trials.

Ideally, while conducting a pharmacokinetic trial, data are obtained that best reflect clinical practice. For drugs that are taken once daily by cancer patients, data on the absolute bioavailability data at steady state plasma pharmacokinetics best reflect clinical drug use. For all investigated smPKIs, the absolute bioavailability trials were performed as single‐dose studies. Furthermore, only 4 trials determined absolute bioavailability in cancer patients.^24^, ^26^, ^27^ An absolute bioavailability trial following multiple dosing in healthy volunteers to achieve steady‐state conditions may not be possible because of ethical considerations. The SILM approach would allow for implementation of investigations at steady‐state plasma pharmacokinetics in cancer patients, with only a mild increase in burden for the patient as a result of administration of the intravenous microdose. The administration of an intravenous microdose during a typical phase 1/2 trial is an easy and straightforward way to assess the absolute bioavailability at steady‐state plasma pharmacokinetics in patients. We recently demonstrated the usefulness of this approach in an absolute bioavailability microdose trial in cancer patients on long‐term imatinib treatment.^28^ Because of this potential, studies that would have previously only been performed in healthy volunteers in the future may be conducted in cancer patients, resulting in more clinically relevant data.

By further implementation of isotopic tracer techniques for the assessment of absolute bioavailability, important gaps in knowledge can be filled with only minor adaptations required to current clinical study designs. Future research can expand to other areas as well, for example, pharmacokinetic studies to investigate any possible changes in pharmacokinetic parameters in special patient populations. The use of improved pharmacokinetic trial designs is especially relevant, as more oral anticancer agents are expected to be developed in the coming years. Only for the group of smPKIs, there are currently already about 175 different orally effective protein kinase inhibitors in clinical trials worldwide.^16^ Advances in SILM research might aid in simplifying study procedures and may result in both increased knowledge on drug pharmacokinetics and a reduction in costs and time associated with the clinical drug development. This combination of factors makes it likely that microdose approaches in combination with LC‐MS/MS will become more important in the future, as they have great not yet fully exploited potential.

## Conclusion

In conclusion, we found that for more than half the oral smPKIs currently on the market, an absolute bioavailability trial was not performed. The SILM trial design provides a useful approach for conducting absolute bioavailability trials in a more effective way and to generate results with increased validity. The emergence of ultrasensitive triple quadrupole LC‐MS/MS technologies allows for more straightforward trial procedures compared with 2‐period crossover trials and ^14^C‐radiolabeled microdosing trials, paving the way for a more widespread implementation of the SILM approach. For all investigated smPKIs, a SILM trial was considered feasible, but only for 2 drugs was this approach actually used during clinical development. With the increase in the number of oral anticancer agents in clinical development, the stable isotopically labeled microdose strategy may alleviate pressure on the investigation of pharmacokinetic parameters by reducing the amount of resources required as well as patient burden. Our review therefore demonstrates that the use of isotopically labeled drugs in clinical drug development provides many not yet fully exploited possibilities to improve absolute bioavailability trial design in oncology.

## Conflicts of Interest

The authors declare no conflict of interest in relation to this study.

## Supporting information

Additional supplemental information can be found by clicking the Supplements link in the PDF toolbar or the Supplemental Information section at the end of web‐based version of this article.
