# Supplementary material for: Stable Isotopically Labeled Intravenous Microdose Pharmacokinetic Trials as a Tool to Assess Absolute Bioavailability: Feasibility and Paradigm to Apply for Protein Kinase Inhibitors in Oncology
Source: Clin Pharmacol Drug Dev. 2020 Jun 22;9(5):552–9. doi: 10.1002/cpdd.840 (PMC7383911; doi:10.1002/cpdd.840)
Supplement: Supplementary file 1 — Additional supplemental information can be found by clicking the Supplements link in the PDF toolbar or the Supplemental Information section at the end of web‐based version of this article. [file CPDD-9-552-s001.docx]

**Supplementary Table 1.** FDA-approved protein kinase inhibitors in oncology for which an absolute bioavailability trial was performed (n=21).

| **Drug** | **Initial year approved** | **Subjects** | **Subjects (n)** | **Study type** | **Oral dose** | **Intravenous dose** | **F (%)** | **References** |
| --- | --- | --- | --- | --- | --- | --- | --- | --- |
| abemaciclib | 2017 | Healthy volunteers | 11 | Stable isotopically labeled microdose | 200 mg | 0.4 mg  (^13^C_8_-labeled) | 45 | ^1–3^ |
| acalabrutinib | 2017 | Healthy volunteers | 8 | Radiolabeled microdose | 100 mg | <10 µg (≤ 1 µCi) | 25 | ^4^ |
| alectinib | 2015 | Healthy volunteers | 6 | Radiolabeled microdose | 600 mg | 50 µg | 37 | ^5^ |
| axitinib | 2012 | Healthy volunteers | 16 | Two-period crossover | 5 mg | 1 mg | 58 | ^2^ |
| bosutinib | 2012 | Healthy volunteers | 7 | Two-period crossover | 100 mg | 120 mg | 34 | ^6,2^ |
| cobimetinib | 2015 | Healthy volunteers | 13 | Two-period crossover | 20 mg | 2 mg | 46 | ^7,8^ |
| crizotinib | 2011 | Healthy volunteers | 14 | Two-period crossover | 250 mg | 50 mg | 43 | ^9,2^ |
| dabrafenib | 2013 | Cancer patients | 4 | Radiolabeled microdose | 150 mg | 50 µg (200 nCi) | 95 | ^10,2^ |
| dacomitinib | 2018 | Healthy volunteers | 14 | Two-period crossover | 45 mg | 20 mg | 80 | ^2^ |
| erlotinib | 2004 | Healthy volunteers | 62* | Two-period crossover | 150 mg | 25 mg | 59 | ^11,12^ |
| gefitinib | 2003 | Healthy volunteers & cancer patients | 12 & 19 | Two-period crossover | 250 mg | 50/100 mg | 57 & 59 | ^2^ |
| ibrutinib | 2013 | Healthy volunteers | 8 | Stable isotopically labeled microdose | 560 mg | 100 ug  (^13^C_6_-labeled) | 4 | ^2,13^ |
| imatinib | 2001 | Healthy volunteers | 12 | Two-period crossover | 400 mg | 100 mg | 98 | ^14,15^ |
| larotrectinib | 2018 | Healthy volunteers | 12 | Radiolabeled microdose | 100 mg | 7.5 ug | 34 | ^2^ |
| lorlatinib | 2018 | Healthy volunteers | Unknown | Two-period crossover | 100 mg | 50 mg | 81 | ^2^ |
| nintedanib | 2014 | Healthy volunteers | 30** | Two-period crossover | 100 mg | 6 mg | 5 | ^16,17^ |
| osimertinib | 2015 | Healthy volunteers | 10 | Radiolabeled microdose | 80 mg | 100 µg (1 µCi) | 70 | ^18,2^ |
| palbociclib | 2015 | Healthy volunteers | Unknown | Two-period crossover | 125 mg | Unknown | 46 | ^2^ |
| pazopanib | 2009 | Cancer patients | 7 | Two-period crossover | 800 mg | 5 mg | 21 | ^19^ |
| trametinib | 2013 | Cancer patients | 4 | Radiolabeled microdose | 2 mg | 5 µg | 72 | ^20,2^ |
| vemurafenib | 2011 | Cancer patients | 6 | Radiolabeled microdose | 960 mg | 20 µg | 58 | ^21^ |

* Part of bioequivalence study, ** Multiple doses investigated

Abbreviations: *F,* absolute bioavailability*; N/A,* not applicable

**References**

1. Turner K, Chappell J, Kulanthaivel P, Ng WT, Royalty J. Abstract CT152: Food effect on the pharmacokinetics of 200-mg abemaciclib in healthy subject. *Cancer Res*. 2016;76(14 Suppl)

2. U.S. Food and Drug Administration - Clinical Pharmacology and Biopharmaceutics Review. Webpage: <https://www.accessdata.fda.gov/scripts/cder/daf/>

3. Robert M, Frenel JS, Bourbouloux E, et al. Pharmacokinetic drug evaluation of abemaciclib for advanced breast cancer. *Expert Opin Drug Metab Toxicol*. 2019;15:85-91

4. Podoll T, Pearson PG, Evarts J, et al. Bioavailability, biotransformation, and excretion of the covalent Bruton tyrosine kinase inhibitor acalabrutinib in rats, dogs, and humans S. *Drug Metab Dispos*. 2019:145-154.

5. Morcos PN, Yu L, Bogman K, et al. Absorption, distribution, metabolism and excretion (ADME) of the ALK inhibitor alectinib: results from an absolute bioavailability and mass balance study in healthy subjects. *Xenobiotica*. 2017;47:217-229

6. Hsyu PH, Pignataro DS, Matschke K. Absolute Bioavailability of Bosutinib in Healthy Subjects From an Open-Label, Randomized, 2-Period Crossover Study. *Clin Pharmacol Drug Dev*. 2018;7:373-381

7. Musib L, Choo E, Deng Y, et al. Absolute bioavailability and effect of formulation change, food, or elevated pH with rabeprazole on cobimetinib absorption in healthy subjects. *Mol Pharm*. 2013;10:4046-4054

8. Takahashi RH, Choo EF, Ma S, et al. Absorption, metabolism, excretion, and the contribution of intestinal metabolism to the oral disposition of [14C]cobimetinib, a MEK inhibitor, in humans. *Drug Metab Dispos*. 2016;44:28-39

9. Xu H, O’Gorman M, Boutros T, et al. Evaluation of crizotinib absolute bioavailability, the bioequivalence of three oral formulations, and the effect of food on crizotinib pharmacokinetics in healthy subjects. *J Clin Pharmacol*. 2015;55:104-113

10. Denton CL, Minthorn E, Carson SW, et al. Concomitant oral and intravenous pharmacokinetics of dabrafenib, a BRAF inhibitor, in patients with BRAF V600 mutation-positive solid tumors. *J Clin Pharmacol*. 2013;53:955-961

11. Frohna P, Lu J, Eppler S, et al. Evaluation of the absolute oral bioavailability and bioequivalence of erlotinib, an inhibitor of the epidermal growth factor receptor tyrosine kinase, in a randomized, crossover study in healthy subjects. *J Clin Pharmacol*. 2006;46:282-290

12. Ling J, Johnson KA, Miao Z, et al. Metabolism and excretion of erlotinib, a small molecule inhibitor of epidermal growth factor receptor tyrosine kinase, in healthy male volunteers. *Drug Metab Dispos*. 2006;34:420-426

13. De Vries R, Smit JW, Hellemans P, et al. Stable isotope-labelled intravenous microdose for absolute bioavailability and effect of grapefruit juice on ibrutinib in healthy adults. *Br J Clin Pharmacol*. 2016;81:235-245

14. Peng B, Dutreix C, Mehring G, et al. Absolute Bioavailability of Imatinib (Glivec®) Orally versus Intravenous Infusion. *J Clin Pharmacol*. 2004;44:158-162

15. Gschwind HP, Pfaar U, Waldmeier F, et al. Metabolism and disposition of imatinib mesylate in healthy volunteers. *Drug Metab Dispos*. 2005;33:1503-1512

16. Dallinger C, Trommeshauser D, Marzin K, et al. Pharmacokinetic Properties of Nintedanib in Healthy Volunteers and Patients With Advanced Cancer. J Clin Pharmacol. 2016:1387-1394

17. Stopfer P, Rathgen K, Bischoff D, et al. Pharmacokinetics and metabolism of BIBF 1120 after oral dosing to healthy male volunteers. *Xenobiotica*. 2011;41:297-311

18. Vishwanathan K, So K, Thomas K, Bramley A, English S, Collier J. Absolute Bioavailability of Osimertinib in Healthy Adults. *Clin Pharmacol Drug Dev*. 2019;8:198-207

19. Deng Y, Sychterz C, Suttle AB, et al. Bioavailability, metabolism and disposition of oral pazopanib in patients with advanced cancer. *Xenobiotica*. 2013;43:443-453

20. Leonowens C, Pendry C, Bauman J, et al. Concomitant oral and intravenous pharmacokinetics of trametinib, a MEK inhibitor, in subjects with solid tumours. *Br J Clin Pharmacol*. 2014;78(3):524-532

21. Zhang W, Colburn D, Simmons B, et al. Absolute Bioavailability of Vemurafenib in Patients With BRAF V600 Mutation–Positive Malignancies . *Clin Pharmacol Drug Dev*. 2020;9:496-504
